# Caloric Restriction Suppresses Microglial Activation and Prevents Neuroapoptosis Following Cortical Injury in Rats

**DOI:** 10.1371/journal.pone.0037215

**Published:** 2012-05-15

**Authors:** Natasa Loncarevic-Vasiljkovic, Vesna Pesic, Smilja Todorovic, Jelena Popic, Kosara Smiljanic, Desanka Milanovic, Sabera Ruzdijic, Selma Kanazir

**Affiliations:** Department of Neurobiology, Institute for Biological Research, University of Belgrade, Belgrade, Republic of Serbia; Massachusetts General Hospital and Harvard Medical School, United States of America

## Abstract

Traumatic brain injury (TBI) is a widespread cause of death and a major source of adult disability. Subsequent pathological events occurring in the brain after TBI, referred to as secondary injury, continue to damage surrounding tissue resulting in substantial neuronal loss. One of the hallmarks of the secondary injury process is microglial activation resulting in increased cytokine production. Notwithstanding that recent studies demonstrated that caloric restriction (CR) lasting several months prior to an acute TBI exhibits neuroprotective properties, understanding how exactly CR influences secondary injury is still unclear. The goal of the present study was to examine whether CR (50% of daily food intake for 3 months) alleviates the effects of secondary injury on neuronal loss following cortical stab injury (CSI). To this end, we examined the effects of CR on the microglial activation, tumor necrosis factor-α (TNF-α) and caspase-3 expression in the ipsilateral (injured) cortex of the adult rats during the recovery period (from 2 to 28 days) after injury. Our results demonstrate that CR prior to CSI suppresses microglial activation, induction of TNF-α and caspase-3, as well as neurodegeneration following injury. These results indicate that CR strongly attenuates the effects of secondary injury, thus suggesting that CR may increase the successful outcome following TBI.

## Introduction

Traumatic injury to the brain triggers a complex series of events that can be divided into two phases: primary and secondary injury. Primary injury is the result of immediate mechanical damage to the brain tissue, which results in the irreversible destruction of neural structures. Damage caused by the primary injury is further augmented by the subsequent pathological processes encompassing the secondary injury. Secondary injury evolves over a period of minutes to hours and days, even to several weeks following initial trauma [reviewed in 1 and 2] and it consists of a multifaceted cascade of inflammatory processes that ultimately lead to neuronal degeneration and apoptosis [3]. One of the hallmarks of secondary injury is the increased number of microglial cells and their activation around the injured area [4].

In response to CNS injury, microglia become active and induce detrimental neurotoxic effects by releasing a diverse set of cytotoxic substances, including proinflammatory cytokine TNF-α [5], [6]. TNF-α plays a key role in many physiological and pathological processes including acute and chronic inflammation, and apoptosis [7]. Accordingly, TNF-α produced by microglia is generally thought to induce sequential activation of caspases ultimately resulting in the apoptosis [8], [9]. Amongst caspases, activated caspase-3 is directly linked to the neuronal cell death following TBI [10].

Although TBI is one of the leading causes of death and disability in the human population, treatment strategies for TBI patients are currently limited. Since the initial loss of neurons caused by primary injury cannot be prevented, the main strategy of most brain injury therapies is focused on suppressing deleterious effects of the secondary injury. One of the current approaches is directed towards preventive life-style changes that could lessen the severity of poor outcomes in the event of TBI. It has been shown that different dietary restriction regiments, applied for several months prior to an acute insult, may have neuroprotective properties and could improve post-injury recovery [11]–[14]. Although the molecular underpinnings are still obscure, it is proposed that CR protects the brain by targeting excitotoxicity, inflammation and apoptosis [15], all of which are constitutive processes of secondary injury.

Previous work in our laboratory has demonstrated that exposing animals to CR significantly reduced astrogliosis and modulated neuronal plasticity in response to cortical stab injury [16]. Given that processes of recovery and remodeling are directly dependent on the extent of secondary injury, the goal of this study was to further examine whether 3-month long CR applied prior to CSI could alleviate the severity of secondary injury. Thus, the effects of CR on microglial activation, TNF-α and caspase-3 induction, and the secondary neuroapoptosis were investigated. Considering that these processes play a crucial role in pathogenesis of TBI, the ability to control them can potentially reverse the harmful effects of injury. Our data reveal that CR prior to mechanical cortical injury suppresses microglial activation, TNF-α induction as well as initiation and execution of apoptotic cascade.

## Materials and Methods

### Animals

Adult male Wistar rats (3 months old, weighing 250–300 g) were used in this study. The animals were housed under standard conditions (23±2°C, 60–70% relative humidity, 12 h light and dark cycles; lights were switched on at 07:00; free access to food and water; n = 3 per cage). All animal procedures were approved by the Committee for Ethical Animal Care and Use of the Institute for Biological Research, University of Belgrade (Permit Number: 26/06), which acts in accordance with the NIH Guide for the Care and Use of Laboratory Animals, (NIH Publication No. 85/23).

### Dietary restriction

Daily food intake for each animal was measured for five consecutive days before the beginning of the experiment, and the average daily food intake was calculated as weight of standard laboratory chow pellets per day per animal. The animals (3 months old) were divided into two groups, *ad libitum* (AL; n = 40) and calorie restricted (CR; n = 40). The AL group had unlimited access to food during the entire experiment. Animals from the CR group were fed 50% of the average daily food intake of the AL group, as previously described [16]. Animals were on CR for 3 months prior to surgery. Considering that food demand increases during animal life, daily food intake was measured in the AL group monthly in order to assure that the correct amount of food was provided to the CR rats. Body weight of AL and CR rats was measured every other week. Rats in the CR group were maintained on a restricted feeding regimen during the entire period of recovery following surgery. Food was provided at 12:00. Both groups of animals had *ad libitum* access to water.

### Surgery

Surgery was performed when the rats were 6 months old. Prior to surgery, the animals were anesthetized with an intraperitoneal injection of Nembutal (Serva) in the dose of 50 mg/kg body weight. A cortical stab injury to the left somatosensory cortex was performed as previously described [16]. After surgery, the animals were kept in isolation for 3 h until completely recovered from anesthesia, and were subsequently returned to their home cages in order to avoid social isolation stress (n = 3 per cage). The minimal number of animals was used and all efforts were made to minimize animal suffering.

### Experimental procedure

Animals from both AL and CR groups were randomly divided into five groups (n = 8 per group). The AL and CR animals were subjected to the surgery and groups were formed according to the duration of the recovery period, i.e. 2, 7, 14 and 28 days post injury (dpi). Physiological controls (designated as control) for both groups were not subjected to surgery. At the end of recovery period animals were decapitated, the brains were removed and the entire ipsilateral cortex was collected from each animal for subsequent protein analysis (n = 5 per group). For histological studies, the brains were fixed overnight at 4°C in 4% (w/v) paraformaldehyde (PFA) in PBS (n = 3 per group).

### Western blot analysis

For Western blot analysis, tissue was homogenized with a Dounce homogenizer in 10 vol (w/v) of RIPA buffer (50 mM Tris-Cl pH 7.5, 150 mM NaCl, 1% NP-40, 0.1% SDS, 10 mM EDTA, 10 mM EGTA, 0.5% Triton X-100). Homogenates were cleared by centrifugation (21,000 rcf, 30 min, 4°C), and the supernatants were collected. Protein concentrations were determined using the Micro BCA Protein Assay Kit (Pierce Biotechnology). Equal amounts of proteins (20 µg per lane) were separated by 10% SDS-PAGE. After being transferred onto nitrocellulose membranes (Amersham Bioscience), the membranes were blocked in 5% non-fat dry milk in TBST (50 mM Tris pH 7.4, 150 mM NaCl and 0.05% Tween20) for 1 h at room temperature (RT), and incubated with the primary antibodies. The membranes were incubated with either goat anti-TNF-α (M-18; 1∶1000; Santa Cruz Biotechnology, USA) overnight at 4°C or rabbit anti-caspase-3 antibody that recognizes the 17-kDa cleavage product (1∶500; Cell Signaling Technology, USA) for 2 hours at RT, both in TBST. Subsequently, the membranes were washed in TBST and incubated with the Horse Radish Peroxidase (HRP)-conjugated anti-goat (1∶5000) or anti-rabbit (1∶2000) antibody (Santa Cruz, Biotechnology, USA) in TBST for 1 h at RT. Each blot was re-probed with rabbit anti-actin antibody (1∶10000; Santa Cruz Biotechnology, USA) in TBST for 1 h at RT. The signal was detected by enhanced chemiluminescence (ECL, Amersham Bioscience, USA) and subsequently exposed on an X-ray film.

### Histological analysis

For histochemical analysis, rat brains were cryoprotected with incubations in graded sucrose solutions (10–30%) in 0.01 M phosphate-buffered saline (PBS) (for 24 h at 4°C each). The brains were frozen in isopentane, cooled on dry ice and stored at −80°C. Every fifth coronal section (18 µm thick) through the site of injury was mounted on slides, allowed to dry overnight and stored at −20°C. Up to this point the section preparation was the same for both immunohistochemical analysis and Fluoro-Jade B/Hoechst staining.

#### Immunohistochemical analysis of microglia – Iba1 immunoreactivity

Before immunoreaction the sections were rinsed in 0.01 M PBS and treated with a 0.3% hydrogen peroxide in 20% methanol solution for 30 minutes at room temperature to block endogenous peroxidase. Three washes were performed with 0.01 M PBS between each two consecutive steps during the process. To block nonspecific immunostaining, sections were treated with 3% BSA (Bovine Serum Albumine) in 0.01 M PBS for 1 h at RT. After that, sections were immunostained with mouse anti-Iba-1 (ionized calcium binding adaptor molecule 1) antibody (1∶500, Abcam, UK) in 0.1% Triton X-100/0.01 M PBS overnight at 4°C. Following three washes in 0.01 M PBS, sections were incubated with the HRP labeled secondary anti-goat antibody (1∶200, Dako, Denmark) in 0.01 M PBS overnight at 4°C. Bound antibodies were visualized with DAB (3,3-diaminobenzidine tetrahydrochloride; Dako, Denmark) by the avidin-biotin peroxidase complex method, following standard protocols (Vector Laboratories, Burlingame, CA). All sections were dehydrated in graded ethanol, cleared in xylene, mounted in Canada balsam (Merck, USA). To test the specificity of the reaction a negative control slide was treated in the same way with the omission of the primary antibody, and run alongside the other samples. Images were captured on an Axio Observer Microscope Z1 using an AxioVision4.6 software system (Carl Zeiss, Germany) at a magnification of 20× and 40×.

#### Quantification of Iba1-positive microglial cells

**Figure 1:**
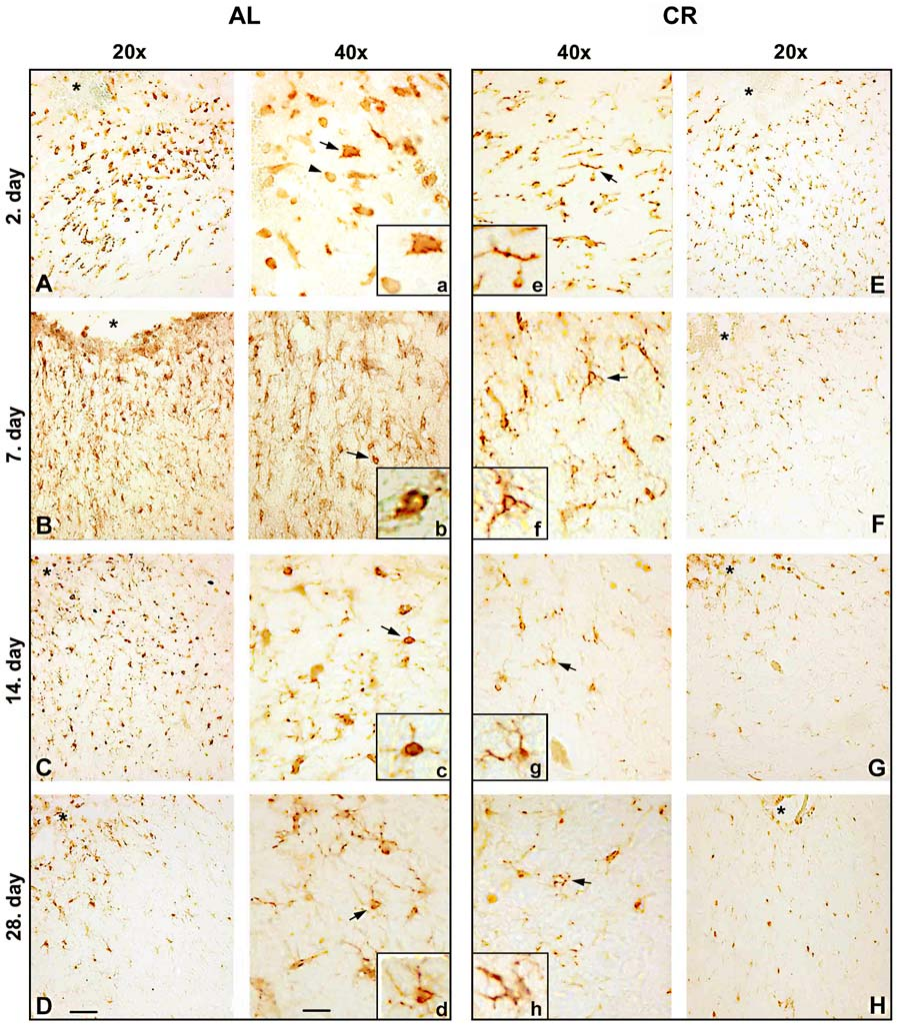
CR suppresses activation of microglia in the perilesioned area when applied prior to cortical stab injury. Brain sections from AL and CR animals 2, 7, 14, 28 days after injury were stained for the Iba-1 marker of microglial cells. Activated microglial cells displaying highly amoeboid and round-shaped morphology (Fig. 1a) were observed only in the AL group on the 2^nd^ day following injury (Fig. 1A). A number of moderately activated microglial cells (Fig. 1b, c) were observed in AL animals on the 7^th^ and 14^th^ day following injury (Fig. 1B, C). In the CR group microglial cells retained a mainly ramified structure throughout the recovery period (Fig. 1 E–H). Images are representative of 3 animals per experimental group; asterisk – site of the lesion. Scale bars: 50 µm and 25 µm for 20× and 40× magnification, respectively.

Rating scale providing an evaluation of microgliosis based on the morphological and immunoreactivity changes was used for quantitative analysis of Iba1-positive microglial cells (Table 1). The morphology of microglial cells was scored according to 3 point categorical rating criteria. Three cellular profiles were defined based on to the length and thickness of their processes and the characteristics of their cell body. Representative examples of each cell type are illustrated in Fig. 1 (insets).

**Table 1:** Description of the qualitative scoring system for microglia activation state.

Activation state	Criteria
**Resting** (Fig. 1e)	Ramified cells with fine processes
**Moderate** (Fig. 1b)	Microglia displaying shortened and thickened processes
**Intense** (Fig. 1a)	Microglia displaying hypertrophy of cell bodies and retraction of processes, with apparent amoeboid or round-shape morphology

For the counting of microglial cells, total of three cryostat coronal sections cut at 18 µm (separated by 90 µm intervals) were used from each brain. Total of 3 brains was used for each time point following injury. For each brain section three rectangles (200 µm×200 µm) were superimposed over the image covering left, right and the area bellow the lesion. Iba1-positive microglial cells in resting, moderate and intense activation state were counted for each rectangle of each section by the blinder observer.

#### Fluoro-Jade B and Hoechst 33258 staining

Fluoro-Jade B staining was performed in order to visualize neuronal cells undergoing degeneration and cell death, while Hoechst 33258 staining was used to confirm neuronal cell death by examining the state of chromatin. Slides were first immersed in a basic alcohol solution consisting of 1% NaOH in 80% ethanol, distilled water and incubated in 0.06% KMnO4 solution for 10 min. Afterwards they were transferred for 10 min to a 0.0001% solution of Fluoro-Jade B (Chemicon International, USA) dissolved in 0.1% acetic acid. Slides were then rinsed by three changes of distilled water for 1 min per change, immersed in 0.01% Hoechst 33258 (Acros Organics, USA) staining solution for 10 min and cover slipped with glycerol. The sections were examined with an Axio Observer Microscope Z1 (Carl Zeiss, Germany) using a filter system suitable for visualizing fluorescein isothiocyanate. Cells labeled with Fluoro-Jade B were observed as individual, shiny green spots that were clearly discernible from the background. The number of degenerating neurons labeled by Fluoro-Jade B and Hoechst staining was counted in 3 fields under the area of the screen (0.38 mm^2^) in 5 sections per animal in the region around the injury (n = 3 animals per each time point for both AL and CR group). The sections were analyzed by a researcher unaware of the treatment.

### Statistical analysis

Significant differences between the sets of data obtained by the counting microglial cells were determined using STATISTICA software, Version 6.0, StatSoft. Student's t-test was used for statistical analysis. Statistical significance was set at p<0.05.

## Results

### CR suppresses microglial activation after CSI

Activation of microglia characterized by changes in their morphology is the hallmark of secondary injury. To determine the effects of CR on microglial response to injury, we characterized the morphology of microglia in the vicinity of the lesion site in both AL and CR animals. To this end, brain sections were immunostained for microglia-specific marker Iba-1 throughout the recovery period (2^nd^, 7^th^, 14^th^ and 28^th^ day) following the initial injury. A large number of Iba-1 positive cells were seen in both AL and CR animals at the site of injury (Fig. 1). The majority of microglial cells seen in the AL animals displayed large round cell bodies, characteristic of the reactive type of microglial cells (Fig. 1A–D). In stark contrast, in the group of animals exposed to CR prior to injury, the microglial cells surrounding the lesion site maintained ramified morphology during the entire recovery period (Fig. 1E–H). The most prominent differences in microglial morphology between AL and CR group were observed on the 2^nd^ dpi, with numerous activated microglial cells with the large amoeboid or round-shape cell bodies observed in AL group (Fig. 1A and E). In control groups of both AL and CR animals, all observed microglial cells had ramified morphology (Fig. S1). All observed microglial cells in the homotypical cortex to the injured area in the contralateral hemisphere of both AL and CR animals had ramified morphology (Fig. S2).

In order to define the level of microglial activation, the number of Iba-1 positive cells according to their activation state was calculated using rating scale (Materials and Methods, Table 1), and obtained results are presented in Table 2. Three cellular profiles for microglial cells were characterized according to the length and thickness of their processes and the characteristics of their cell body (*resting*, *moderate* and *intense*). Total number of microglial cells around the injured site was significantly increased in both AL (Table 2, p<0.05) and CR (Table 2, p<0.05) animals following injury, as compared to appropriate controls. In AL group, activated microglia was observed during entire recovery period with the greatest number of *intense* microglial cells observed at 2^nd^ dpi. The number of *resting* microglia at this time point was significantly lower as compared to appropriate AL control (p<0.05). In CR group by far the greatest number of microglial cells observed around the injury retained resting morphology, while only a few activated, but still *moderate*, cells were observed at 2^nd^ dpi. At the same time, the number of *resting* microglial cells was significantly higher as compared to appropriate CR control (p<0.05). As for the other time points, the number of moderately activated microglia was significantly increased in AL group at 7^th^, 14^th^ and 28^th^ dpi as compared to CR group (p<0.05). The number of intensely activated microglia was significantly increased in AL group at 2^nd^ and 7^th^ dpi as compared to CR group (p<0.05). It is very important to emphasize that, within the AL group, the number of *intense* microglia was by far the highest at the 2^nd^ dpi (p<0.05), while it decreased significantly at later time points following injury. Immunohistochemical analysis did not reveal any conspicuous difference in microglial number between control, non-injured, AL and CR rats.

**Table 2:** Number of microglial cells.

Microglial cells		control	2. day	7. day	14. day	28. day
**Resting**(Fig. 1d–h)	**AL**	9.87±0.81*	4.44±0.93*	16.67±1.12*	22.11±1.05*	11.78±0.60*
	**CR**	10.33±1.03#	28.00±1.09#	21.78±1.10#	14.44±1.21#	11.22±1.10
**Moderate**(Fig. 1b)	**AL**	0	4.11±0.56	9±0.87 $	11.21±1.09 $	2.78±0.43 $
	**CR**	0	3.33±0.53	2.56±0.58	1.33±0.33	0.33±0.17
**Intense**(Fig. 1a)	**AL**	0	17.9±0.91 **^$&^**	2.56±0.43 $	1.44±0.38	0.89±0.35
	**CR**	0	0.56±0.24	0.44±0.18	0.58±0.21	0.33±0.19
**Total number**	**AL**	9.87±0.81	26.44±1.54*	28.2±1.57* $	34.8±1.04* $	15.5±0.53* $
	**CR**	10.33±1.03	31.88±0.74# $	24.77±1.02#	16.33±1.24#	11.89±1.01

Number of Iba-1 positive cells classified according to their morphology in the perilesioned area 2,7,14 and 28 days following SCI in AL and CR animals. Data are expressed as means ± S.E.M. (n = 3 animals per experimental group).

* p<0.05 vs. AL control;

# p<0.05 vs. CR control;

$ p<0.05 AL vs. CR;

& p<0.05 vs. other time points of AL.

**Figure 2:**
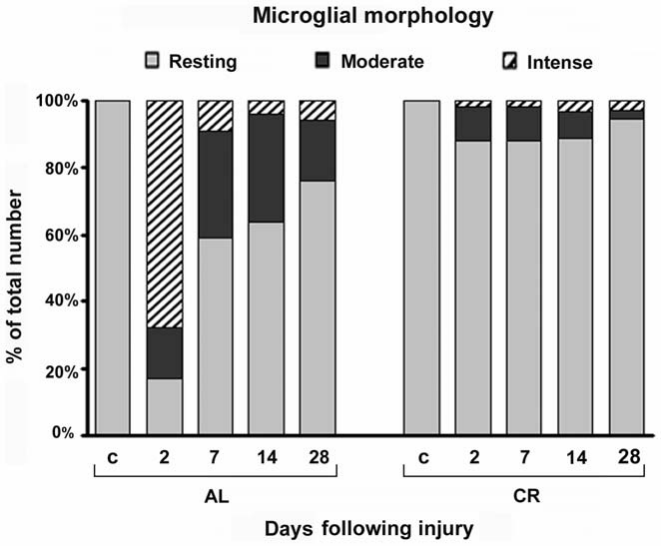
CR modulates microglial morphology in the perilesioned area. Quantification of each morphological type of microglial cell (Table 2) is depicted as the percentage distribution per AL and CR groups as a function of time following injury. Percentage of both *intense* and *moderate* microglial cells was much higher in the AL group compared to the CR group at all time points. By far the greatest percentage of *intense* microglial cells was observed in the AL group on the 2^nd^ day following injury (83%). In the CR group the percentage of activated microglia (both *intense* and *moderate*) did not exceed 15% of the microglia population. c – physiological control; 2, 7, 14, 28 – days following injury.

Analysis of percentage of different microglial activation states (Fig. 2) clearly shows that microglial activation is more pronounced in AL as compared to CR group during entire recovery period following injury. The highest activation rate was observed in the AL group on the 2^nd^ dpi where the activated microglial cells constitute over 80% of the microglial population. Although, the share of activated microglial cells in AL animals decreases significantly over time, it still constitutes over 20% of the population. However, in CR group percentage of activated microglial cells does not exceed 15% of the microglial population during entire recovery period following injury. Taken together, this data indicates that CR strongly attenuates microglial activation in the cortical tissue following mechanical injury.

### CR inhibits induction of soluble TNF-α and active caspase-3 protein in the ipsilateral cortex of CSI rats

**Figure 3:**
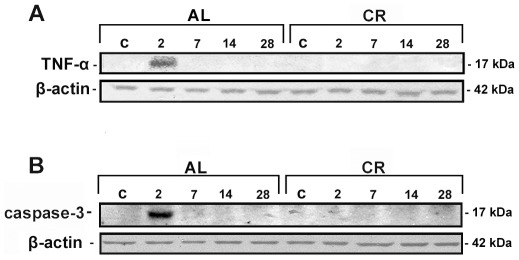
CR treatment inhibits the induction of soluble TNF-α and active caspase-3. Western blot analysis of the temporal expression of soluble TNF-α (A) and active caspase-3 (B) revealed the induction of both proteins only in the ipsilateral cortex of AL animals on the 2^nd^ day following injury. Expression of both proteins was not detected in the CR group. Actin bands are the loading control. Representative immunoblots (n = 5 animals per experimental group) are presented; c – physiological control; 2, 7, 14, 28 – days following injury.

Activated microglial cells are a predominant source of proinflammatory cytokines, particularly TNF-α at the site of injury. Western blot analysis was performed in order to investigate changes in the levels of active form of the TNF-α protein – soluble TNF-α (17 kDa), in the ipsilateral cortex of AL and CR rats at 2^nd^, 7^th^, 14^th^ and 28^th^ dpi (Fig. 3A). Strong induction of soluble TNF-α protein was observed in AL group on the 2^nd^ dpi, whereas soluble TNF-α protein was undetectable at later time points. Remarkably, CR completely suppressed TNF-α induction after injury. Furthermore, active form of TNF-α protein was not detected during the entire recovery period in animals exposed to caloric restriction. These findings demonstrate that CR abolishes TNF-α production induced by CSI.

Since elevated levels of TNF-α protein are known to initiate the caspase-dependent apoptosis [17], we next investigated the changes in the expression profile of activated caspase-3 (17 kDa) after CSI in the ipsilateral cortex of AL and CR animals (Fig. 3B). Western blot analysis revealed that active caspase-3 is strongly induced in the AL group on the 2^nd^ dpi, but not at later time points, which mirrors elevation of soluble TNF-α level. Importantly, CR completely suppressed the induction of active caspase-3. Thus, CR diminishes the effects of CSI on the upregulation in the active caspase-3 levels, which correlates with the attenuation of TNF-α induction.

### CR prevents apoptosis after CSI

**Figure 4:**
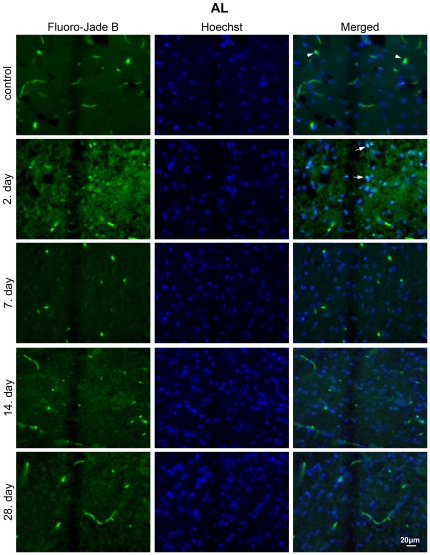
Neuroapoptosis is present in the perilesioned area of AL animals. Fluoro-Jade B and Hoechst staining of brain sections from AL animals 2, 7, 14, 28 days after injury. Numerous Fluoro-Jade B/Hoechst positive cells (merged) were observed in the AL group on the 2^nd^ day following injury (arrows); these cells were not detected at later time points. Images are representative of brain sections at the site of the lesion (n = 3 animals per experimental group); c – physiological control; 2, 7, 14, 28 – days following injury; blood vessels (arrowheads); magnification 40×.

**Figure 5:**
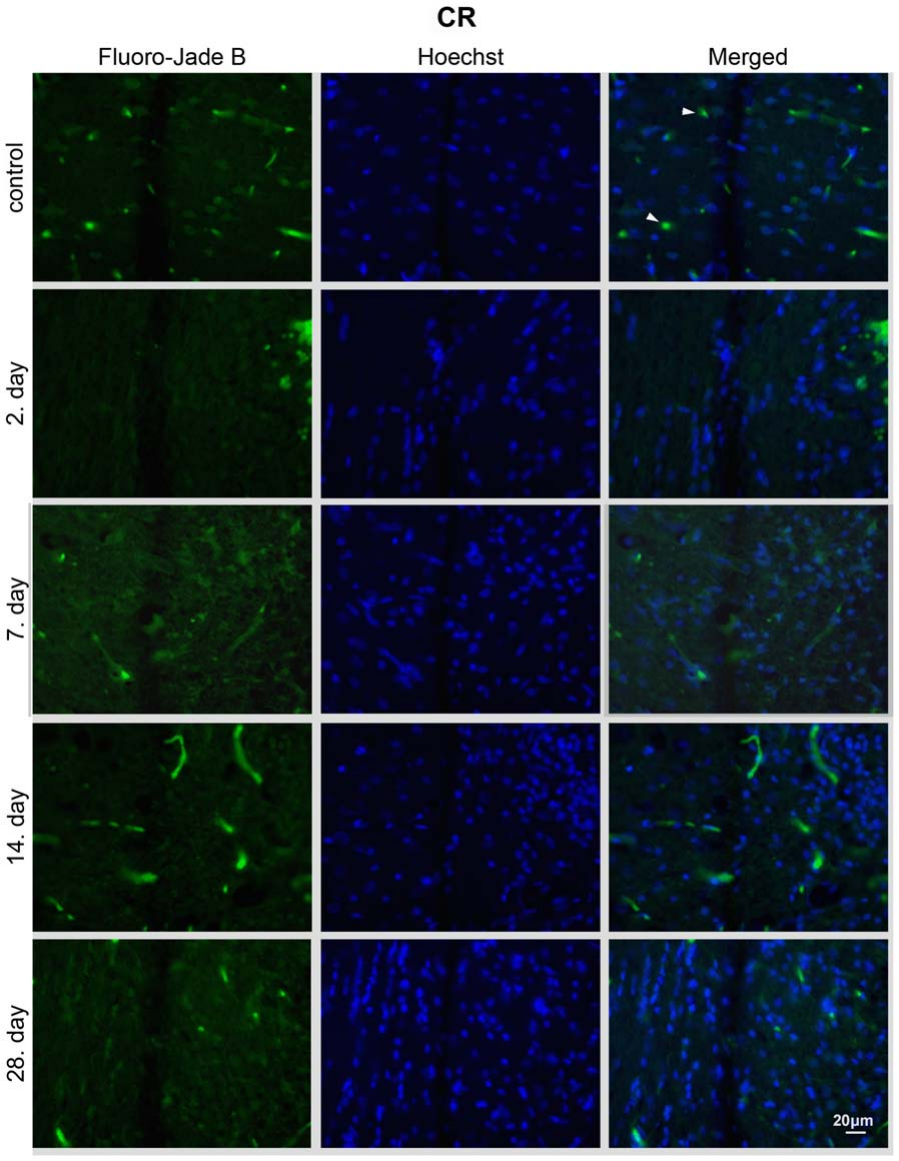
CR treatment suppresses neuroapoptosis in the perilesioned area. Fluoro-Jade B and Hoechst staining of brain sections from CR animals 2, 7, 14, 28 – days after injury. Fluoro-Jade B/Hoechst positive cells were not detected in the CR group at any time point. Images are representative of brain sections at the site of the lesion (n = 3 animals per experimental group); c – physiological control; 2, 7, 14, 28 – days after the injury; blood vessels (arrowheads); magnification 40×.

Because elevated levels of active caspase-3 lead to neurodegeneration and neuronal cell death, Fluoro-Jade B/Hoechst double staining was performed on brain sections from AL (Fig. 4) and CR (Fig. 5) animals following injury. A number Fluoro-Jade B positive cells, an average of 25, was observed only in AL group on the 2^nd^ dpi in the vicinity of the lesion (Fig. 4; arrows), whereas Fluoro-Jade B positive cells were not detected at later time points in AL group. Hoechst staining of nuclei showed chromatin condensation in the nuclei of Fluoro-Jade B positive cells which is indicative of neurodegeneration. Analysis of brain sections of CR animals did not reveal the presence of Fluoro-Jade B positive cells at any point in time, while Hoechst staining displayed a normal chromatin structure of the nuclei observed within the sections (Fig. 5). Staining of the brain sections from control animals did not reveal the presence of Fluoro-Jade B positive cells in either AL or CR group. Fluoro-Jade B positive cells were not observed in the homotypical cortex to the injured area in the contralateral hemisphere of neither AL nor CR animals (Fig. S3).

## Discussion

This study revealed that restricted feeding regimen used prior to injury potently alleviated the deleterious effects of secondary injury by suppressing microglial activation, TNF-α and caspase-3 induction and neuronal cell death following cortical stab injury in rats.

In the normal central nervous system (CNS), microglial cells are highly ramified, with an elaborate tertiary and quaternary branch structure [18]. The highly branched resting microglia provides the brain with a dynamic and efficient surveillance system. Virtually any CNS pathology or damage will lead to their activation and loss of the resting phenotype [18], [19]. Although microglial activation represents an integral part of the CNS response to injury, it is still not clear whether activated microglia promote neuronal survival, or whether these cells further exacerbate the extent of neuronal damage. While some findings imply a supportive role for microglial cells in the induction of neuroplastic changes after ischemia [20], a large body of data has rather convincingly shown that microglia possess neurotoxic properties [reviewed in 21 and 22]. This was supported by the fact that immunosuppressive strategies result in an inhibition of microglial activation and neuroprotection after acute traumatic or ischemic brain or spinal cord injury [19], [23].

Results presented in this study show that, even though a total number of microglial cells around the site of lesion increase significantly in both AL and CR group, morphology of these cells is strikingly different. Namely, the majority of microglial cells seen early after injury in AL animals displayed highly activated morphology with large round cell bodies, whereas in the group of animals exposed to CR prior to injury, the microglial cells surrounding the lesion site maintained ramified morphology during the entire recovery period. While the study of Lee et al. [24] showed that dietary restrictions decreased the number of newly generated microglia following kainate-induced brain lesions, the present study is, to the best of our knowledge, the first to demonstrate that CR, prior to mechanical trauma to the brain, has led to suppression of microglial activation following injury. This result is in good keeping with previous findings which indicate that microglial activation and recruitment, but not proliferation, mediate neurodegeneration following injury [25].

The activated microglia plays a pivotal role in the inflammatory response that lasts hours to days following TBI. Activated microglial cells are the main source of proinflammatory cytokines within the CNS [26]. One of the predominant cytokines secreted by the microglial cells is TNF-α [27]. It has been shown that the TNF-α expression and secretion are rapidly increased in neurons up to 4 hrs following excitotoxic events at the synapse [28]. TNF-α induces proliferation of surrounding microglial cells and stimulates their activation. Importantly, microglial cells continue to express TNF-α as part of the maintenance and amplification of the inflammatory cascade 2–5 days following injury [28]. Accordingly, the present study revealed that strong TNF-α induction occurred in the injured cortex of AL animals on the second day following injury. This correlates with the uppermost number of highly activated microglial cells detected in the overall microglial population. Therewithal, in the animals maintained on caloric restriction the abolishment of TNF-α protein induction was accompanied by a minimal microglial activation rate. Given that the TNF-α represents one of the main pro-inflammatory cytokines involved in initiation and expansion of secondary injury, abolishment of TNF-α protein expression following injury may reduce the extent of secondary injury.

Inflammatory processes following trauma ultimately lead to neuronal degeneration and apoptosis [3]. Active caspase-3 represents a key executor of apoptosis [29], and a major underlying factor responsible for apoptotic cell death following CNS injury [30]. As in other CNS injury paradigms [reviewed in 31], strong induction of active caspase-3 together with numerous degenerating neurons was observed in the injured cortex early after stab injury. Results presented in this study undoubtedly showed that CR represents very potent neuroprotective factor, since it completely abolished the induction of active caspase-3 and neurodegeneration caused by injury. Considering that outcome following injury is directly related to the number of lost neurons, neuronal cell death represents a major issue associated with TBI in the clinic. Thus, many strategies have been developed in an attempt to minimize neuronal cell loss following brain injury. Caloric restriction proved to be neuroprotective by preventing neurons from secondary cell death after mechanical injury.

Most of the studies on the anti-inflammatory and anti-apoptotic mechanisms of CR were focused on the prevention of stroke and other cardiovascular diseases in aging and obesity or in slowing aging processes [32]–[36]. Some recent studies shown that prophylactic CR suppresses systemic inflammation in spontaneously hypertensive rats, and led to a delay in the onset of stroke [37]. However, data concerning effects of CR on processes of secondary injury following stroke, mechanical or some other type of injury, are lacking. Our study is to the best of our knowledge the first to show that prophylactic CR suppresses injury-induced microglial activation, active caspase-3 induction and neuronal cell death in the injured rat cortex, consistent with the inhibitory effect of fasting on ischemia-induced increases of TNF-α [38]. Even though the exact mechanisms of neuroprotective properties of CR remain unknown, it is tempting to speculate that CR might be capable of mimicking the immunosuppressive action of drugs in reducing damage following brain trauma.

Although the paradigm of pre-injury caloric restriction may appear as a treatment with limited clinical relevance, recent data showed that CR is more effective in improving functional recovery if applied pre- than post-injury [39]. It is likely that cellular pathways for neuroprotection have been already activated by pre-injury CR (days to months before injury), and that this provides benefits during the early secondary post-injury phase. Therefore, if the diet is applied after TBI, we can assume that beneficial CR-induced effects will occur too slowly to influence the early cascades of secondary injury (hours to days).

In conclusion, our data provide evidence that CR ameliorates secondary injury after CSI by repressing microglial activation, TNF-α production, caspase-3 activation and neuronal cell death. These results, together with our previous report [16] strongly suggest that prophylactic CR, as a part of a preventive life style could lead to better outcomes following brain injury.

## Supporting Information

Figure S1 **Morphology of microglial cells of AL and CR control animals.** Iba-1 staining of the ipsilateral cortex of AL and CR control animals. Microglial cells of AL and CR groups display the same ramified morphology. Representative sections at 20× and 40× magnification (n = 3 animals per experimental group).
(TIF)

Figure S2 **Morphology of microglial cells in the contralateral hemisphere of AL and CR animals.** Representative sections showing Iba-1-staining of the homotypical cortex to the injured area in the contralateral hemisphere, in both AL and CR animals on the 2nd day following injury. Microglial cells of AL and CR groups display the same, ramified morphology. Representative sections at 20× magnification (n = 3 animals per experimental group).
(TIF)

Figure S3 **Fluoro-Jade B/Hoechst staining of the contralateral hemisphere of AL and CR animals.** Representative sections showing Fluoro-Jade B/Hoechst staining of the homotypical cortex to the injured area in the contralateral hemisphere, in both AL and CR animals on the 2nd day following injury. Degenerating neurons were not observed in brain sections of AL nor CR animals. Images are representative of brain sections at the site of the lesion (n = 3 animals per experimental group). Blood vessels (arrowheads); magnification 20×.
(TIF)
