# Supplementary figures and images for: Caloric Restriction Suppresses Microglial Activation and Prevents Neuroapoptosis Following Cortical Injury in Rats
Source: PLoS One. 2012 May 15;7(5):e37215. doi: 10.1371/journal.pone.0037215 (PMC3352891; doi:10.1371/journal.pone.0037215)

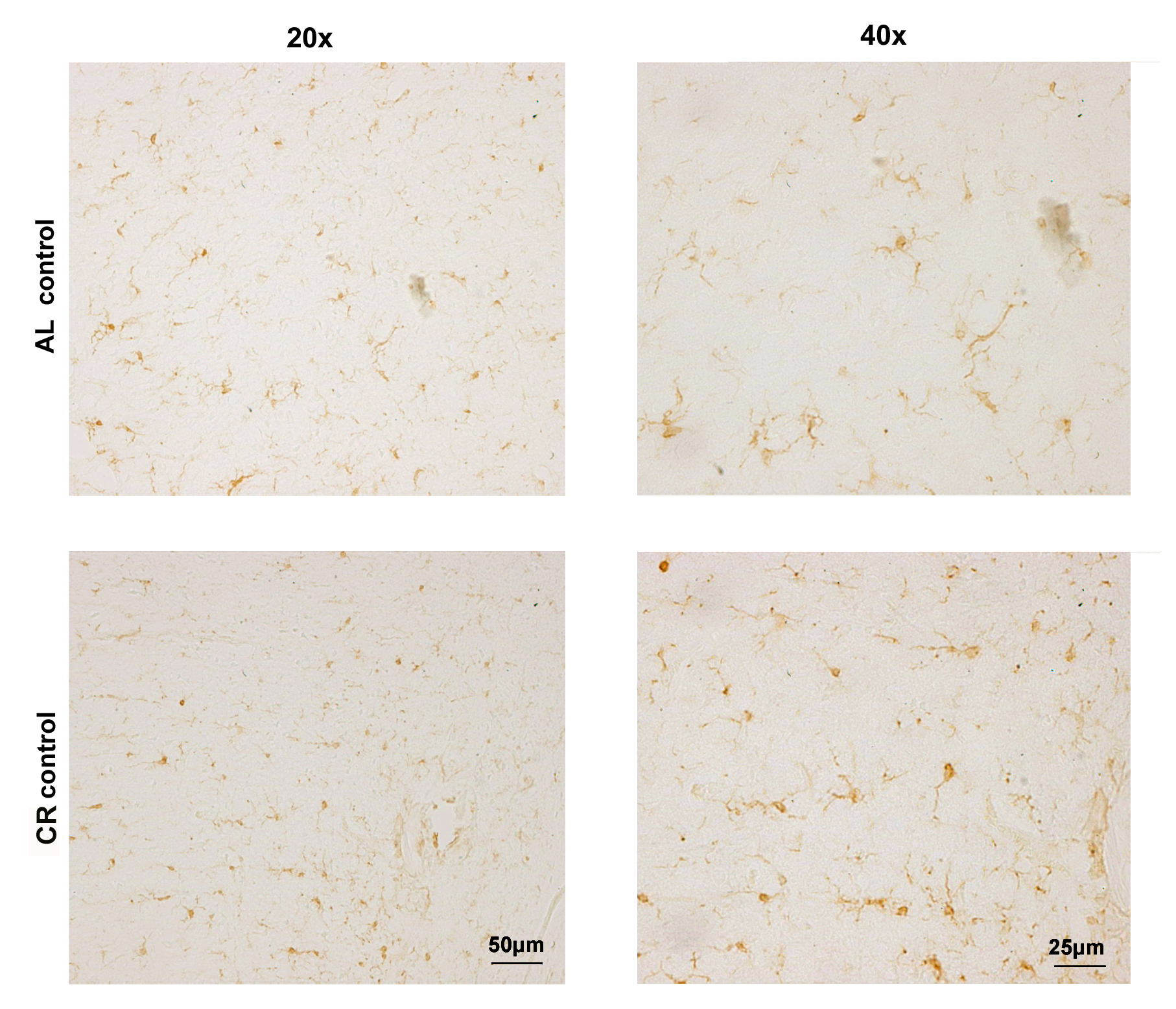

Supplement: Figure S1 — Morphology of microglial cells of AL and CR control animals. Iba-1 staining of the ipsilateral cortex of AL and CR control animals. Microglial cells of AL and CR groups display the same ramified morphology. Representative sections at 20× and 40× magnification (n = 3 animals per experimental group). (TIF) [file pone.0037215.s001.tif]

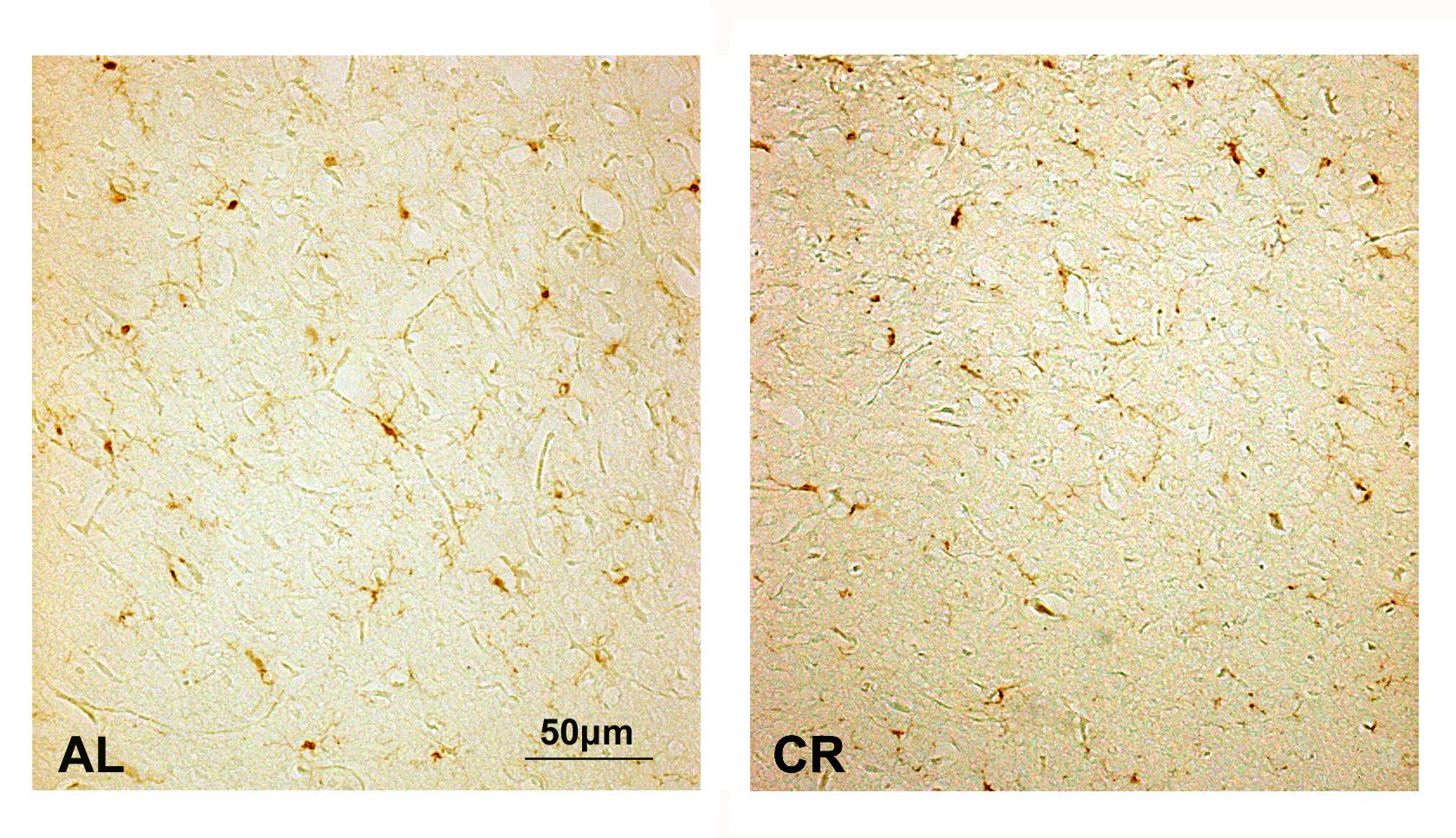

Supplement: Figure S2 — Morphology of microglial cells in the contralateral hemisphere of AL and CR animals. Representative sections showing Iba-1-staining of the homotypical cortex to the injured area in the contralateral hemisphere, in both AL and CR animals on the 2nd day following injury. Microglial cells of AL and CR groups display the same, ramified morphology. Representative sections at 20× magnification (n = 3 animals per experimental group). (TIF) [file pone.0037215.s002.tif]

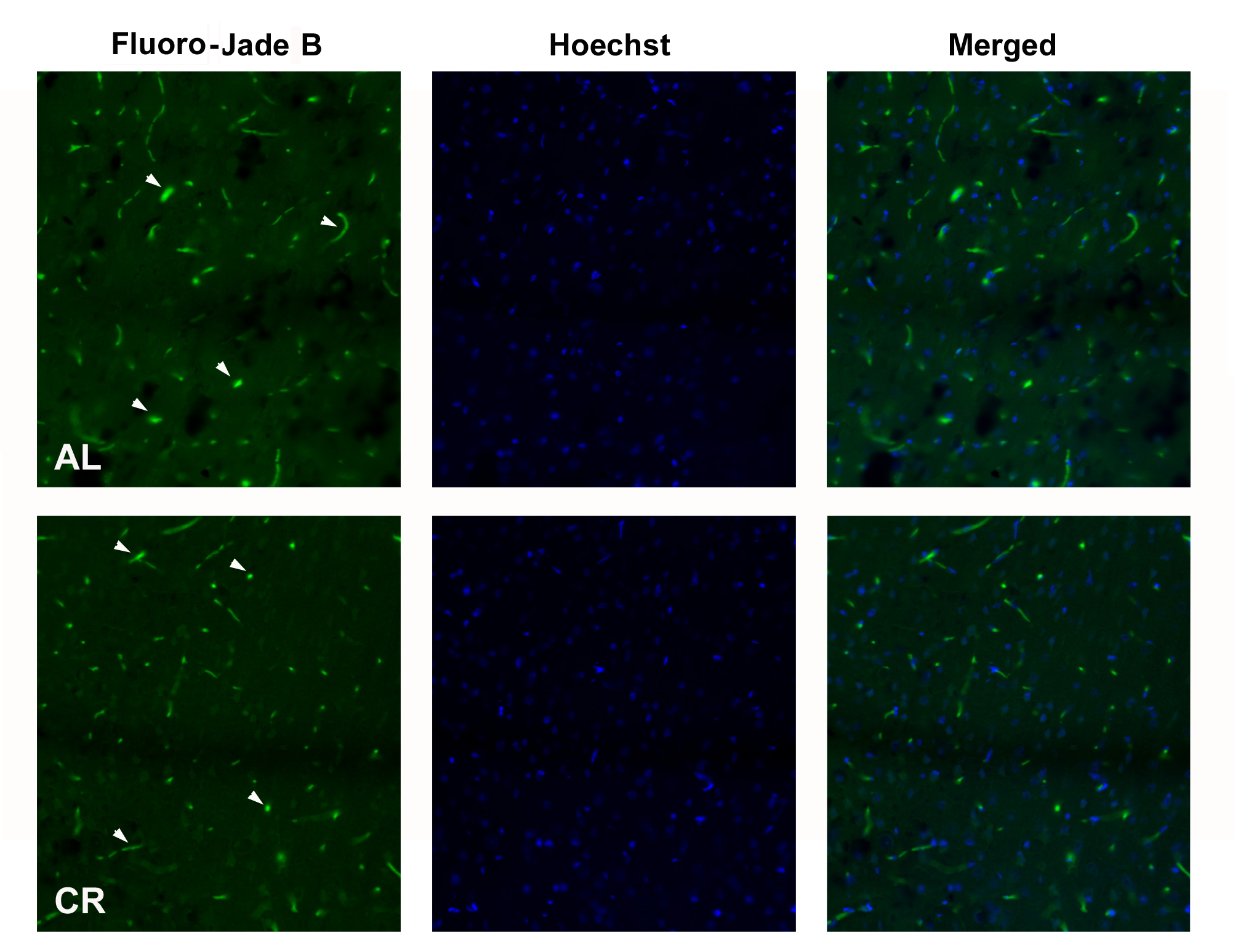

Supplement: Figure S3 — Fluoro-Jade B/Hoechst staining of the contralateral hemisphere of AL and CR animals. Representative sections showing Fluoro-Jade B/Hoechst staining of the homotypical cortex to the injured area in the contralateral hemisphere, in both AL and CR animals on the 2nd day following injury. Degenerating neurons were not observed in brain sections of AL nor CR animals. Images are representative of brain sections at the site of the lesion (n = 3 animals per experimental group). Blood vessels (arrowheads); magnification 20×. (TIF) [file pone.0037215.s003.tif]
